# Factors influencing the health-related quality of life of Chinese advanced cancer patients and their spousal caregivers: a cross-sectional study

**DOI:** 10.1186/s12904-016-0142-3

**Published:** 2016-08-02

**Authors:** Qiuping Li, Yinghua Xu, Huiya Zhou, Alice Yuen Loke

**Affiliations:** 1 Wuxi Medical School, Jiangnan University, Wuxi, Jiangsu Province China; 2 Wuxi People’s Hospital, Wuxi, Jiangsu Province China; 3 School of Nursing, The Hong Kong Polytechnic University, Hung Hom, Kowloon Hong Kong, China

**Keywords:** Advanced cancer, Spousal caregivers, Caregiver-patient dyads, Health-related quality of life, SF-12, Depression, Anxiety, Chinese

## Abstract

**Background:**

Cancer and its treatment have a major impact on the lives of patients and their intimate partners, such as on their health-related quality of life (HRQOL). The aims of this study are to: (i) assess the HRQOL of advanced cancer patients and spousal caregivers, and explore the relationship between the HRQOL of cancer patients and that of their spousal caregivers; (ii) detect factors influencing the HRQOL of cancer patients and spousal caregivers; and (iii) explore the impact of anxiety and depression on the HRQOL of couples.

**Methods:**

A total of 131 couples where one of the partners was hospitalized for advanced cancer were invited to complete a survey to assess their demographic and background information, HRQOL, and anxiety and depression. HRQOL was measured using the SF-12, while anxiety and depression were measured using the Hospital Anxiety and Depression Scale. Data were analyzed using a *T*-test, Pearson correlations, multiple linear regressions, and structural equation modeling.

**Results:**

In general, the spousal caregivers had higher levels of HRQOL (seven out of eight SF-12 domains and two SF-12 dimensions) *p* = 0.038–0.000, anxiety (*p* = 0.002), and depression (*p* = 0.011) than patients. Correlations of HRQOL between patients and spouses were small to moderate (*r* = 0.193–0.398). Multiple independent factors influencing the physical component summary (PCS), mental component summary (MCS), vitality (VT), and role emotional (RE) sections of the SF-12 were identified, including: gender, time since diagnosis, levels of education, working status, the extent to which spousal caregivers were informed about the disease, improved marital relationship after the diagnosis of cancer, and anxiety and depression. For both patients and spousal caregivers, the strongest independent factor influencing HRQOL (SF-12 PCS, MCS, VT, and RE) was anxiety and depression. Anxiety and depression may have both actor and partner effects on the HRQOL of couples to various degrees.

**Conclusions:**

The findings of this study call attention to the HRQOL of couples and its influencing factors. Individual characteristics of cancer patients and spouses, marital relationship, and anxiety and depression are highlighted as areas in which couples coping with cancer could benefit from interventions to improve their HRQOL.

**Electronic supplementary material:**

The online version of this article (doi:10.1186/s12904-016-0142-3) contains supplementary material, which is available to authorized users.

## Background

Cancers are among the leading causes of morbidity and mortality worldwide. In 2012, there were 14.1 million new cases of cancer and 8.2 million deaths from cancer worldwide. Of these, 21 % of the new cancer cases (3 million) and 27 % of the cancer deaths (2.2 million) occurred in China [1]. The World Health Organization (WHO) expects the number of new cases of cancer to rise by about 70 % over the next two decades [1]. It is unfortunate that in developing countries, where treatment options are both limited and expensive, most new cases of cancer are frequently diagnosed at the advanced stage [2]. This late diagnosis of cancer and the limited treatment options available in developing countries, e.g., in China, may lead to poor prognoses for cancer, which in turn may affect the health-related quality of life (HRQOL) of cancer patients. This further supports the argument that more attention should be paid to the problem of the HRQOL of cancer patients. The challenges posed by cancer and its treatment are felt not only by patients but also by the members of their family [3], particularly by spouses, who are typically the primary caregivers of cancer patients [4].

Thus, these challenges must be addressed on the individual and dyadic levels. A diagnosis of cancer is a crisis for individuals, who are confronted not only with the cancer and its treatment, but also faced with the possibility of physical disability, threats to their family and social roles and relationships, and concerns about life and death [5]. Spouses also face challenges such as worries about their ability to provide emotional and practical support, and the potential loss of their life partner from cancer [6, 7]. Both patients and their spouses also need to cope together as dyads along the cancer trajectory. Couples go through a process of readjusting and adapting, which includes breaking the “bad news” to other family members, managing household and childcare responsibilities, negotiating changes in family and social roles, and interferences with life plans [6, 8, 9]. A review of the mutual impact between the spousal caregiver and the cancer patient revealed that the process of coping with cancer affects both parties, with reciprocal influences and congruence between the spousal caregiver-patient dyads [10].

The challenges posed by the diagnosis and treatment of cancer, and the changes in the relational dynamics between cancer patients and their spouses, can have an impact on the HRQOL of both patients and their spouses [11]. Each partner’s HRQOL can also affect that of the other [12, 13]. HRQOL is a multidimensional concept encompassing perceptions of both the negative and positive aspects of the dimensions of physical, emotional, social, and cognitive function [14]. In cancer practice, the measure of HRQOL can supply valuable information on the health status and effects of treatment on patients and spousal caregivers [15], and can be adopted in clinical care as a predictor of positive coping [16].

The experience of couples coping with cancer together, however, is complex and relationships are dynamic [17]. The mutual impact between couples, in terms of HRQOL, and the factors influencing HRQOL have not been well delineated, particularly from the perspective of the dyadic level in mainland China. It is essential to address this issue, if the HRQOL of both cancer patients and their spousal caregivers is to be optimized.

Most studies in this area have focused on the HRQOL of patients and its influencing factors [18–22]. Various factors have been reported as influencing the QOL of patients, including the patients’ socio-demographic characteristics, such as age, gender, working status, family income, level of education [18–21], and symptom distress (e.g., anxiety and depression) [19, 20, 23, 24]. However, which factors influence the HRQOL of cancer patients remains inconclusive. For example, a study on cancer patients undergoing chemotherapy reported that demographic variables, e.g., age, education, marital status, or income, were not significantly related to the QOL of the patients [22].

A study exploring the factors influencing the QOL of spousal caregivers in China showed that the QOL of spousal caregivers was associated with demographic characteristics of the spouses, caregiving-related variables, and the severity of the patients’ symptoms [25]. However, another study revealed contradictory results, with gender, level of education, and time spent on caring found to be not associated with the QOL of the family caregivers of cancer patients [26]. Studies have also revealed that the relationship between the couples also influences the QOL of the spousal caregivers of cancer patients [26, 27].

There have been very few studies on the factors influencing the HRQOL of couples from the dyadic perspective. A longitudinal study that assessed the QOL of both prostate cancer patients and their spouses showed that better QOL for both partners was associated with the following factors: a relatively low level of education on the part of patient, older age on the part of partner, a higher family income, and localized cancer at baseline [14]. Another study reported that the overall QOL of both patients and caregivers was influenced by the following factors: the cancer diagnosis, length of hospitalization, caregiving intensity and duration, marital satisfaction, and caregiving self-esteem [4].

These studies revealed that the factors influencing the HRQOL of couples coping with cancer potentially include socio-demographic characteristics, spousal or marital relationship, and symptoms of distress. Thus, to achieve a better understanding of the HRQOL of couples coping with cancer and the factors influencing it, variables such as the characteristics of the patients and spousal caregivers, the characteristics of the married couples, and their anxiety and depression were selected as potential influencing factors in the present study.

To our knowledge, no studies have been conducted in mainland China assessing the HRQOL and associated factors of both advanced cancer patients and their spousal caregivers. Thus, this study is the first of its kind. The specific objectives of this study are to: (i) assess the HRQOL of patients with advanced cancer and their spousal caregivers, and the relationship between the HRQOL of the advanced cancer patients and that of their spousal caregivers; (ii) identify factors influencing the HRQOL of advanced cancer patients and their spousal caregivers; and (iii) explore the impact of anxiety and depression on the couples’ HRQOL. This study will shed light on the HRQOL of advanced cancer patients and their spousal caregivers and their mutual impact, providing evidence for developing interventions to improve the HRQOL of couples.

## Methods

### Study design and subjects

This was a cross-sectional observational study. Advanced cancer patients and their spousal caregivers were recruited from November 2013 to July 2014 from a hospital in the city of Wuxi, China.

The criteria for couples to be considered for inclusion in the study were: (i) Chinese adult married couples (age >18 years old); (ii) a medical diagnosis of any type of advanced cancer in one partner; (iii) the spouse is the primary caregiver, who provides informal care to the cancer patient; (iv) both partners agreed to take part in the study. The spousal caregiver was defined and identified by the cancer patient as his or her married partner, and was the primary source of physical and emotional support since the diagnosis of cancer. Excluded from the study were couples with the following characteristics: (i) cancer patients who had other major health problems, such as dementia; (ii) spousal caregivers who were unable to care for patients due to chronic illness or who were suffering from a serious physical or mental illness, including cancer; and (iii) those who were unable to communicate in Mandarin (the language commonly spoken in China).

The sample size was calculated using G-power 3.1.9.2, based on the conventional method of analyzing power for correlations [28]. Assuming a two-sided type I error of 5 %, with 90 % power, and a medium effect size (*r* = 0.30) [29] to detect the resulting correlation in paired observations (cancer patients and spousal caregivers), it was estimated that at least 112 dyads would be required. To ensure an adequate number of samples, 150 couples were approached.

Of the 150 eligible couples who were approached, 19 declined. The reasons given by the couples for refusing to participate in the study were that they were not interested in the study (*n* = 8), they were too busy (*n* = 6), or they did not want to complete a questionnaire (*n* = 2). Three couples refused to give a reason. As a result, a total of 131 couples were included in the study.

### Instruments

A questionnaire was compiled based on existing tools, including the Medical Outcomes Study 12-item Short Form (MOS SF-12) (version 2) for HRQOL [30] and the Hospital Anxiety and Depression Scale (HADS) for Anxiety and Depression [31].

A demographic and background information sheet was used to collect information on socio-demographic characteristics and clinical data from both the patients and their spousal caregivers. The socio-demographic data included: age, gender, level of education, working status, duration of marriage, perceived relationship with one’s partner before the diagnosis of cancer, if there were perceived changes in the relationship with one’s partner after the diagnosis of cancer, and if there was a financial burden on the family due to the cancer-related treatment. The clinical data included: type of cancer, stage of cancer, time since diagnosis, the extent to which the couple has been informed about the diagnosis of cancer, and the amount of time that the spousal caregiver is spending in caring for the patient (hours/day).

Health-related quality of life (HRQOL) was measured using the Medical Outcomes Study 12-item Short Form (MOS SF-12) (version 2) [30]. SF-12 consists of 12 items making up eight scales that measure the following eight domains of HRQOL: Physical Functioning (PF), Role-Physical (RP), Bodily Pain (BP), General Health (GH), Vitality (VT), Role-Emotional (RE), Social Functioning (SF), and Mental Health (MH). The eight SF-12 domains hypothetically form two dimensions: the Physical Component Summary (PCS) and the Mental Component Summary (MCS). The PCS includes the four scales of PF, RP, BP, and GH; while the MCS contains the other four scales of VT, RE, SF, and MH [30]. The eight scales and the two dimensions were transformed and calculated according to the SF-12 (version 2) score manual to a scale with a theoretical range of 0 to 100 [32]. A higher score indicates a higher level of HRQOL. The two dimensions (PCS and MCS) of the SF-12 version 2 achieved R squares of 0.905 with PCS and 0.938 with MCS when the SF-36 was used in a cross-validated Medical Outcomes Study. Test-retest (2-week) correlations of 0.89 and 0.76 were observed for the SF-12 PCS and MCS respectively in the general U.S. population (*n* = 232) [30].

The original English version of the SF-36 was translated and validated in a Chinese population in China [33]. A health survey study was conducted to determine whether the SF-12 is an equivalent substitute for the SF-36. The Chinese version of the SF-12 (version 2) was established to be a valid, reliable, and sensitive measurement for Chinese populations [34]. The Cronbach's alpha of the Chinese version of the SF-12 was 0.910 in an elderly Chinese population [35].

The anxiety and depression of the patients and spousal caregivers were measured using the Hospital Anxiety and Depression Scale (HADS) [31]. The scale contains 14 items making up two subscales of anxiety and depression, with seven items in each subscale. A four-point Likert-type scale (0–3) is used for scoring each item, and the total score for each subscale is obtained by a simple summation of the individual items. Scores in each subscale (anxiety and depression) range from 0 to 21. The higher the scores, the higher the levels of anxiety and depression. The HADS is a validated and widely used self-reported measure that assesses individuals’ self-perceived levels of anxiety and depression. It is used to identify patients with elevated levels of symptoms and disorders of anxiety and depression that may be clinically relevant. The Cronbach’s alpha for HADS anxiety varied from 0.68 to 0.93 (mean 0.83) and for HADS depression from 0.67 to 0.90 (mean 0.82) [36].

The original English version of HADS was first translated into Chinese and validated in a Hong Kong population [37]. The Chinese version of HADS was proven to have good linguistic, structural, and scale equivalence with the original English version [37]. Its sound psychometric properties demonstrated its appropriateness for use among in-patients [38]. The Cronbach’s alpha for the Chinese version of HADS was 0.86 for the full scale, 0.82 for the depression subscale, and 0.77 for the anxiety subscale [38].

The internal consistency of the two translated instruments in this study among cancer patients and spousal caregivers was acceptable, with a Cronbach’s alpha of 0.81 for SF-12, and a Cronbach’s alpha of 0.85 for both components of HADS on anxiety and depression in cancer patients; and of 0.84 and 0.85 for HADS anxiety and depression respectively among spousal caregivers.

### Data collection procedure

Before the commencement of the study, ethical approval for the study was granted by the research ethics committee of Jiangnan University. Informed written consent was obtained from the participants prior to the study. It was made clear to them that participation was voluntary and that they were free to withdraw from the study at any time for any reason, with no penalty. All of the information provided by the participants would remain confidential and their anonymity would be preserved. Only the members of the study research team could access the data.

Prior to the commencement of the study, nurses working in the oncology unit of the study hospital were provided with explanations of the study and of the instruments. The oncologists in the hospital identified couples in accordance with the eligibility criteria. Couples who met the criteria for inclusion were approached in the oncology wards when they were admitted for chemotherapy treatments. After they were given an explanation of the study and their written informed consent to participate in it was obtained, the couples were invited to complete the questionnaires separately with the help of nurses if needed. The questionnaires were completed in an in-patient private room or nurses’ office, according to the preference of the couples, in order to protect their privacy and keep them away from possible disturbances. The questionnaire took about 10–15 min to complete. None of the participants reported feeling discomfort or distress while filling out the questionnaire.

### Data analysis

The data analysis was performed using the Statistical Package for the Social Sciences, version 21.0 (SPSS, Chicago, Illinois, USA). The level of significance was set *p* < 0.05. Descriptive statistics such as frequencies, percentages, means, and standard deviations were used to describe the characteristics and clinical profile of the participants. Paired *T*-test and Pearson correlations were used to compare differences and correlations between the paired variables of the patients and the spousal caregivers. T-tests, one-way ANOVAs, and Pearson correlations were performed to examine the associations between different domains or dimensions of the SF-12, including the Physical and Mental Components Summary, and potential associated variables, such as gender, age, level of education, type of cancer, and anxiety and depression. The factors influencing HRQOL were analyzed by multiple linear regressions.

Preparing the data for the multiple linear regressions involved the following tasks: (1) creating dummy variables for categorical variables [29], including gender, being informed about the disease, level of education, and working status. For example, for the variable gender, the dummy variable of male vs. female was created by coding male = 1 and female = 0; (2) extracting the principal component for related variables to avoid collinearity between them [29]. For example, to avoid a possible collinearity between anxiety and depression with a high correlation (*r* = 0.83 for both patients and spousal caregivers) in this study, the principal component of anxiety and depression was extracted by conducting a factor analysis. Thus, HAD (the principal component from a factor analysis of anxiety and depression - PCFAC) was used in the following regression analysis.

Structural equation modeling (SEM) guided by the Actor Partner Interdependence Model (APIM) [39] using Amos 21.0 was conducted to explore the impact of anxiety and depression on HRQOL. Thus, APIM analysis was used in this study to specify the SEM analysis. The APIM analysis is considered a versatile approach to modeling dyadic data [39]. In the APIM, actor effect is the effect of an individual’s characteristics (e.g., anxiety) on their own measured outcomes (i.e., PCS); while partner effect refers to the effect of an individual’s characteristics on the measured outcomes of their partner. The maximum likelihood method was applied to estimate the covariance matrices in all of the selected models. Three indices were used to evaluate the goodness of fit of the model, namely: Chi-Square χ^2^, a confirmatory fit index (CFI), and a root mean square error of approximation (RMSEA). The level considered indicative of a good model fit was set at an insignificant p value of *p* > 0.05 in the Chi-Squareχ^2^, a value of above 0.95 in the CFI, and a value of less than 0.08 in the RMSEA [40].

## Results

Table 1 summarizes the characteristics of these couples. Nearly two thirds of the patients (62.6 %) were male. Although over half of the couples (51.5 %) had been diagnosed for less than 6 months, all of the patients were in the advanced stage of cancer, namely, stage III (*n* = 56, 42.7 %) and stage IV (*n* = 75, 57.3 %). More than half of the patients (55.0 %) suffered from a cancer of the digestive system (e.g., esophageal, gastric, liver, or colorectal cancer). The great majority of patients reported that they had a “very good” (87.8 %) relationship before the diagnosis of cancer. Over half of the families (58.8 %) experienced a serious financial burden due to the cost of treating the cancer.

**Table 1:** The characteristics of patients and spousal caregivers

Characteristics	Patients (P, *n*=131)	Spousal caregivers (SC, *n*=131)		
	*n* (%)	*n* (%)		
Age (mean± SD), years	56.6±12.1 (ranging from 25 to 79)	56.0±11.3 (ranging from 29 to 80)		
Gender				
Male	82 (62.6)	49 (37.4)		
Female	49 (37.4)	82 (62.6)		
Level of education				
Primary school or less	64 (48.9)	67 (51.1)		
High school	49 (37.4)	49 (37.4)		
University or above	18 (13.7)	15 (11.5)		
Working status				
Working	75 (57.3)	69 (52.7)		
Not working	56 (42.7)	62 (47.3)		
The average time since diagnosis/the duration in their role as a SC	13.7±23.0 months (ranging from 1 to 192 months)	<6 months:	67 (51.2)	
		6 months ~2 years:	43 (32.8)	
		>2 years ~5 years:	13 (9.9)	
		>5 years:	8 (6.1)	
Couples were informed about the disease^b^				
Partly informed	33 (25.2)	24 (18.3)		
Well informed	98 (74.8)	107 (81.7)		
Time spent by SC in caring for patients/day [in hours, n (%)]		<2 hours:	10 (7.6)	
		2~4 hours:	14 (10.7)	
		>4~6 hours:	25 (19.1)	
		>6~8 hours:	21 (16.0)	
		>8 hours:	61 (46.6)	
Types of cancer^a^	Digestive system cancer: 72 (55.0); Lung cancer: 35 (26.7);			
	Urogenital system cancer: 19 (14.5); Others: 5 (3.8)			
Dyad Characteristics^c^				
Duration of marriage (mean± SD), years	30.8±11.6 (ranging from 1–52)			
Relationship with their partner before the diagnosis of the cancer	Very good Bad	115 (87.8) 0	Normal	16 (12.2)
Change in the relationship with their partner after the diagnosis of the cancer	Improved Getting worse	38 (29.0) 0	No change	93 (71.0)
Financial burden on the family due to the treatment of cancer	Serious	77 (58.8)	Normal	49 (37.4)
	Mild or None	5 (3.8)		

*SD* standard deviation

^a^All of the patients had advanced cancer, were in stage III (*n* = 56, 42.7 %) or stage IV (*n* = 75, 57.3 %); and were receiving chemotherapy

^b^Well informed: The patient fully understood his/her condition; or the SC was well informed about his/her spouse’s disease; Partly informed: The patient was informed about the diagnosis of cancer, but not about the severity of his/her condition; or the SC were partly informed about his/her spouse’s disease

^c^The data for the dyad characteristics were reported by the patients

### HRQOL and HADS correlations and differences between cancer patients and spousal caregivers

Table 2 shows the mean scores and standard deviations scores of two dimensions and eight domains of SF-12, the anxiety and depression scores of HADS, and the correlations and differences between the cancer patients and spousal caregivers. The results show significant correlations in all paired variables between the patients and their spousal caregivers, with the exception of the “role physical” item of SF-12. The effect sizes of the association between the patients’ HRQOL and that of their spouses were small to moderate (r ranged from 0.193 to 0.398). The spousal caregivers had higher scores for most of the subscales of SF-12 (with the exception of MCS, SF, and MH), and higher levels of anxiety and depression than the patients.

**Table 2:** Scores for HRQOL and HADS: paired samples correlations and differences between patients and spousal caregivers

Paired variables	Patients (n = 131)				Spousal caregivers (*n* = 131)				*r*	*P*-value	t	*P*-value
	Minimum	Maximum	Mean	SD	Minimum	Maximum	Mean	SD				
Two dimensions of SF-12												
PCS	16.26	62.82	37.70	9.46	18.61	61.60	43.05	9.13	0.384	0.000^a^	−5.931	0.000^b^
MCS	22.85	65.62	41.92	7.18	25.71	62.39	42.25	7.70	0.398	0.000^a^	−0.472	0.638
Eight domains of SF-12												
Physical Functioning	22.11	65.05	40.14	10.59	22.11	56.47	42.57	10.89	0.241	0.005^a^	−2.098	0.038^b^
Role Physical	20.32	57.18	38.01	8.34	20.32	57.18	41.95	9.28	0.088	0.319	−3.782	0.000^b^
Bodily Pain	16.68	57.44	36.13	13.22	16.68	57.44	42.20	11.54	0.435	0.000^a^	−5.244	0.000^b^
General Health	18.87	61.99	35.44	11.28	18.87	61.99	41.32	12.58	0.308	0.000^a^	−4.778	0.000^b^
Vitality	27.62	67.88	44.75	8.89	27.62	67.88	47.21	9.61	0.245	0.005^a^	−2.473	0.015^b^
Role Emotional	11.35	56.08	34.78	10.68	11.35	56.08	38.11	9.95	0.193	0.027^a^	−2.905	0.004^b^
Social Functioning	16.18	56.57	39.76	9.75	16.18	56.57	40.92	10.72	0.287	0.001^a^	−1.080	0.282
Mental Health	21.87	64.54	44.21	8.67	15.77	64.54	43.65	9.50	0.368	0.000^a^	0.624	0.534
HADS												
Anxiety	0.00	18.00	7.36	4.00	0.00	19.00	8.52	4.17	0.496	0.000^a^	−3.233	0.002^b^
Depression	0.00	21.00	6.89	4.48	0.00	21.00	7.95	4.70	0.470	0.000^a^	−2.587	0.011^b^

*HRQOL* health-related quality of life, *HADS* hospital anxiety and depression scales, *SD* standard deviation, *SF-12* the Medical Outcomes Study 12-item Short Form (MOS SF-12), *PCS* physical component summary, *MCS* mental component summary

^a^Significant difference in the correlations between paired variables

^b^Significant difference in mean scores (paired *T*-test) between paired variables

Table 2 also shows that the scores for vitality (VT, 44.75 ± 8.89 for patients and 47.21 ± 9.61 for spousal caregivers) were the highest and those for role emotional (RE, 34.78 ± 10.68 for patients and 38.11 ± 9.95 for spousal caregivers) were the lowest among all of the eight domains of the SF-12. To better understand the HRQOL of cancer patients and their spousal caregivers, apart from analyzing SF-12 PCS and MCS as is generally done [41], in the present study VT and RE with the highest and lowest scores were selected as the two SF-12 domains used to conduct the following further analysis.

### Factors influencing HRQOL

Additional file 1: Table S1 shows associations between various variables of both patients and spousal caregivers with the patients’ results in the SF-12 domains of PCS, MCS, VT, and RE. These variables are listed under the four sections on the left side of Additional file 1: Table S1 and consist of the patients’ characteristics (S1a), the spousal caregivers’ characteristics (S1b), the dyads’ characteristics (S1c), and the patients’ and spousal caregivers’ HADS scores (S1d). Additional file 1: Table S2 gives the results of associations between the variables of both patients and spousal caregivers with the spousal caregivers’ results in the SF-12 domains of PCS, MCS, VT, and RE. Again, the variables on the left side of the table were also organized under four sections, namely: patients’ characteristics (S2a), spousal caregivers’ characteristics (S2b), dyads’ characteristics (S2c), and HADS (S2d).

Variables that had significant (*p* < 0.05) or borderline (p ≤ 0.086 in this case) significant differences with any of the four SF-12 scores (PCS, MCS, VT, and RE) in Additional file 1: Table S1 and Table S2 were further analyzed as independent variables in the next step to identify the factors influencing HRQOL using multiple linear regressions. The independent variables that were found in the previous analysis to be significant included: (1) patients’ characteristics: gender, time since the diagnosis of cancer; (2) spousal caregivers’ characteristics: level of education, time spent by spousal caregivers in caring for patients per day; (3) dyads’ characteristics: change in the relationship with their partner after the diagnosis of cancer, and financial burden on the family due to the cancer treatment; (4) HADS: anxiety and depression of both the patients and their spousal caregivers. The independent variables that were found in the previous analysis to be borderline significant included: (1) patients’ characteristics: being informed about the disease (RE, *p* = 0.086, PCS, *p* = 0.084); (2) spousal caregivers’ characteristics: working status (VT, *p* = 0.058), being informed about the disease (RE, *p* = 0.078).

Table 3 shows the results of the factors associated with the HRQOL: SF-12 PCS, MCS, VT, and RE scores using multiple linear regressions in patients. The factors that were identified included: (3a) patients’ characteristics: male patients (female spousal caregivers) were associated with a higher SF-12 VT score (*p* = 0.029); a longer duration since the diagnosis of cancer was associated with a lower SF-12 VT score (*p* = 0.020); (3b) spousal caregivers’ characteristics: a primary school level of education was associated with higher scores for SF-12 MCS (*p* = 0.019) and RE (*p* = 0.003); and a high school level of education was associated with a higher SF-12 RE-P score (*p* = 0.036) when compared with a university level of education or above. Working status was associated with a higher SF-12 VT score (*p* = 0.026) than was the case with non-working status. Compared with being well informed about the disease, being partly informed about the disease was associated with higher scores for SF-12 VT (*p* = 0.025) and RE (*p* = 0.035); (3c) dyads’ characteristics: improved dyadic relationship after the diagnosis of cancer was associated with a lower SF-12 RE score (*p* = 0.037) than was the case with those who saw no change in their relationship with their partner; and (3d) HADS: a higher level of anxiety and depression in patients (PCFAC: HAD) was associated with lower scores for SF-12 PCS (*p* = 0.002), MCS (*p* = 0.001), VT (*p* = 0.004), and RE (*p* = 0.000).

**Table 3:** Factors associated with four domains (PCS, MCS, VT, and RE) of the SF-12 of the patients by multiple linear regressions

Independent variables	PCS_P		MCS_P		VT_P		RE-P	
	Coef B (95 % CI)	*P*-value	Coef B (95 % CI)	*P*-value	Coef B (95 % CI)	*P*-value	Coef B (95 % CI)	*P*-value
Constant	44.32(33.47,55.17)	0.000	37.39(29.55,45.23)	0.000	34.68(25.01,44.34)	0.000	32.80(21.80,43.80)	0.000
3a: Patients’ characteristics								
Male vs. Female_P^a^	2.18(−1.23,5.60)	0.207	−0.56(−3.019,1.91)	0.656	3.40(0.36,6.44)	0.029	−1.98(−5.44,1.48)	0.259
Time since diagnosis of cancer	−0.02(−0.09,0.05)	0.533	−0.05(−0.10,0.004)	0.074	−0.07(−0.14,−0.01)	0.020	−0.05(−0.12,0.02)	0.142
Partly vs. Well informed	−1.10(−4.87,2.67)	0.565	−1.95(−4.67,0.78)	0.160	0.35(−3.01,3.70)	0.839	−3.02(−6.84,0.80)	0.120
3b: Spousal caregivers’ characteristics								
Level of education (vs. University or above)								
Primary school	1.06(−4.46,6.57)	0.705	4.79(0.80,8.77)	0.019	3.30(−1.62,8.21)	0.186	8.71(3.12,14.30)	0.003
High school	0.26(−5.14,5.66)	0.925	2.61(−1.29,6.51)	0.187	1.47(−3.34,6.28)	0.546	5.86(0.38,11.33)	0.036
Working vs. Non working	0.83(−2.41,4.07)	0.612	0.93(−1.42,3.27)	0.435	3.28(0.40,6.17)	0.026	0.81(−2.48,4.10)	0.626
Partly vs. Well informed	2.09(−2.18,6.37)	0.334	2.20(−0.89,5.29)	0.161	4.36(0.55, 8.17)	0.025	4.67(0.33, 9.00)	0.035
Time spent in caring for P/day	−0.87(−2.12,0.38)	0.172	0.03(−0.87,0.94)	0.945	−0.02(−1.14,1.09)	0.969	−0.44(−1.71,0.83)	0.491
3c: Dyads’ characteristics								
Change in the relationship with their partner after the diagnosis of cancer								
Improved vs. No change	−0.53(−4.06,2.99)	0.765	−0.91(−3.46,1.64)	0.481	2.90(−0.24,6.04)	0.070	−3.81(−7.39,−0.24)	0.037
Financial burden on the family due to the treatment of cancer (vs. Mild or None)								
Serious	−6.23(−14.87,2.42)	0.157	1.20(−5.05,7.45)	0.704	2.77(−4.30,10.48)	0.477	−1.84(−10.61,6.93)	0.678
Normal	−4.63(−13.39,4.14)	0.298	2.98(−3.36,9.31)	0.354	4.60(−3.21,12.41)	0.245	1.76(−7.13,10.65)	0.696
3d: HADS								
PCFAC: HAD of patients	−3.00(−4.90,−1.10)	0.002	−2.40(−3.77,-1.03)	0.001	−2.49(−4.18,−0.80)	0.004	−3.54(−5.47,−1.61)	0.000
PCFAC: HAD of SC	−0.51(−2.39,1.37)	0.594	−0.80(−2.16,0.56)	0.248	−0.37(−2.05,1.31)	0.662	−1.53(−3.44,0.38)	0.115

*PCS* physical component summary, *MCS* mental component summary, *VT* vitality, *RE* role emotional, *P* patient, *SC* spousal caregivers; *Coef B* unstandardized regression coefficient B; *CI* confidence interval, *PCFAC*: *HAD* principal component from factor analysis of anxiety and depression

^a^The findings in the gender analysis of the patients are the opposite of those in the gender analysis of spousal caregivers

Table 4 displays the results of the factors associated with the HRQOL: SF-12 PCS, MCS, VT, and RE scores using multiple linear regressions in spousal caregivers. Two aspects of factors were identified under the subgroups of dyads’ characteristics (4c) and HADS (4d). In dyads’ characteristics (4c), an improved dyadic relationship after the diagnosis of cancer was associated with a higher score for SF-12 VT (*p* = 0.005) and a lower score for SF-12 RE (*p* = 0.017) when compared with those who saw no change in their relationship with their partner after the diagnosis of cancer. Regarding HADS (4d), a higher level of anxiety and depression in patients (PCFAC: HAD-P) was only associated with a lower SF-12 MCS score (*p* = 0.008); while a higher level of anxiety and depression in spousal caregivers (PCFAC: HAD of SC) was associated with lower scores for the spousal caregivers in the SF-12 domains of PCS (*p* = 0.001), MCS (*p* = 0.001), VT (*p* = 0.020), and RE (*p* = 0.001).

**Table 4:** Factors associated with four domains (PCS, MCS, VT, and RE) of the SF-12 of the spousal caregivers by multiple linear regressions

Independent variables	PCS_SC		MCS_SC		VT_SC		RE-SC	
	Coef B (95 % CI)	*P*-value	Coef B (95 % CI)	*P*-value	Coef B (95 % CI)	*P*-value	Coef B (95 % CI)	*P*-value
Constant	50.36(39.92,60.81)	0.000	43.37(34.92,51.82)	0.000	52.83(41.75,63.92)	0.000	44.20(33.26,55.13)	0.000
4a: Patients’ characteristics								
Male vs. Female_P^a^	0.27(−3.01,3.55)	0.871	0.02(−2.63,2.68)	0.987	0.92(−2.57,4.40)	0.603	1.16(−2.28,4.60)	0.505
Time since diagnosis of cancer	−0.03(−0.09,0.04)	0.458	−0.01(−0.06,0.05)	0.786	−0.01(−0.08,0.06)	0.730	−0.004(−0.07,0.07)	0.906
Partly vs. Well informed	−0.97(−4.60,2.66)	0.598	0.69(−2.25,3.63)	0.643	2.01(−1.84,5.86)	0.303	1.77(−2.03,5.57)	0.357
4b: Spousal caregivers’ characteristics								
Level of education (vs. University or above)								
Primary school	0.48(−4.83,5.79)	0.858	−0.67(−4.96,3.63)	0.758	−1.35(−6.99,4.28)	0.636	1.48(−4.08,7.04)	0.600
High school	0.15(−5.05,5.35)	0.955	−0.51(−4.71,3.70)	0.812	−1.22(−6.74,4.30)	0.663	1.75(−3.69,7.19)	0.525
Working vs. Non working	0.42(−2.70,3.54)	0.790	−0.22(−2.74,2.31)	0.865	0.57(−2.74,3.88)	0.732	0.88(−2.38,4.15)	0.593
Partly vs. Well informed	−1.55(−5.67,2.56)	0.457	1.62(−1.71,4.95)	0.336	1.08(−3.29,5.44)	0.627	−3.29(−7.60,1.02)	0.133
Time spent in caring for P/day	0.09(−1.11,1.30)	0.880	−0.50(−1.48,0.47)	0.310	−0.94(−2.22,0.33)	0.146	−0.36(−1.62,0.91)	0.578
4c: Dyads’ characteristics								
Change in the relationship with their partner after the diagnosis of cancer								
Improved vs. No change	0.22(−3.17,3.62)	0.898	−0.80(−3.55,1.95)	0.565	5.18(1.57,8.78)	0.005	−4.36(−7.92,−0.81)	0.017
Financial burden on the family due to the treatment of cancer (vs. Mild or None)								
Serious	−8.29(−16.62,0.04)	0.061	1.00(−5.73,7.74)	0.769	−4.13(−12.96,4.71)	0.357	−7.54(−16.26,1.17)	0.089
Normal	−7.15(−15.59,1.29)	0.096	1.92(−4.91,8.74)	0.580	−3.59(−12.54,5.37)	0.429	−3.79(−12.63,5.04)	0.397
4d: HADS								
PCFAC: HAD of patients	−0.68(−2.51,1.15)	0.464	−2.00(−3.48,−0.52)	0.008	−0.79(−2.73,1.15)	0.422	−1.33(−3.25,0.58)	0.171
PCFAC: HAD of SC	−3.09(−4.90,−1.27)	0.001	−2.51(−3.97,−1.04)	0.001	−2.29(−4.22,−0.37)	0.020	−3.18(−5.08,−1.29)	0.001

*PCS* physical component summary, *MCS* mental component summary, *VT* vitality, *RE* role emotional, *P* patient; *SC* Spousal Caregivers; *Coef B* Unstandardized regression Coefficient B, *CI* confidence interval, *PCFAC: HAD* principal component from a factor analysis of anxiety and depression

^a^The findings of the gender analysis of the patients are the opposite of those in the gender analysis of the spousal caregivers

### Impact of anxiety and depression on HRQOL

APIM was used to test the impact of anxiety and depression on HRQOL. It was decided that four models will be analyzed using the two theoretical dimensions of SF-12, PCS and MCS [41]; and the two data-driven components that were selected based on the results of this study. Additional file 2: Figure S1 shows the theoretical APIM for testing the impact of anxiety and depression on HRQOL. As shown, anxiety directly or indirectly through depression impacted on the SF-12 domains as actor effects (from A-a to A-d) and/or as partner effects (from P-a to P-d) in the four models, respectively. The SF-12 domains in the four models (Models 1–4) were: PCS, MCS, VT, and RE.

The APIM analysis of all of the four sub-models (Additional file 2: Figure S2 and Table 5) resulted in convergence and showed goodness of fit, with the indices of Chi-Square χ^2^ = 2.345, p = 0.310; CFI =0.999; and RMSEA = 0.036. These good fits to the models of the APIM analysis provide support for the view that anxiety and depression are interrelated and have both actor and partner effects on couples’ health-related quality of life to various degrees.

**Table 5:** Standardized path coefficients and fit statistics of four models

Indicates	M 1	M 2	M 3	M 4
Dyadic variables	PCS	MCS	VT	RE
Number of distinct sample moments:	27	27	27	27
Number of distinct parameters to be estimated	25	25	25	25
Degrees of freedom	2	2	2	2
Anxiety_P Depression_P	0.82**	0.82**	0.82**	0.82**
Anxiety_SC Depression_SC	0.83**	0.83**	0.83**	0.83**
Anxiety _P SDs_P (A-a)	−0.14	−0.32*	−0.02	−0.21
Anxiety _P SDs_SC (P-a)	0.06	−0.09	0.26	0.002
Depression_P SDs_P (A-b)	−0.22	−0.02	−0.23	−0.12
Depression_P SDs_SC (P-b)	−0.16	−0.18	−0.33*	−0.22
Anxiety_SC SDs_SC (A-c)	−0.13	−0.18	−0.08	−0.24
Anxiety_SC SDs_P (P-c)	−0.16	−0.05	−0.11	−0.18
Depression_SC SDs_SC (A-d)	−0.22	−0.16	−0.25	−0.07
Depression_SC SDs_P (P-d)	0.03	−0.12	0.01	−0.03
Chi-square *X* ^2^	2.345	2.345	2.345	2.345
Probability level (*P* > 0.05)	0. 310	0. 310	0. 310	0. 310
a confirmatory fit index (CFI > 0.95)	0.999	0.999	0.999	0.999
a root mean square error of approximation (RMSEA < 0.08)	0.036	0.036	0.036	0.036

*SDs* SF-12 Domains, *P* patients, *SC* spousal caregivers

A-a, A-b, A-c, A-d, A-d stands for Actor effects

P-a, P-b, P-c, P-d stands for Partner effects

M1 to M4 represent four different sub-models. M1: PCS = physical component summary; M2: MCS = mental component summary; M3: VT = Vitality; M4: RE = role emotional

*P < 0.05; **P < 0.01

A further analysis of the four sub-models using APIM analysis found that: (1) in general, the impact of anxiety and depression on HRQOL (PCS, MCS, VT, and RE) was negative, with five exceptions relating to partner effects (Additional file 2: Figure S2 and Table 5), including the impact of the patients’ anxiety on their spouses’ PCS, MCS, and RE; the impact of the spouses’ depression on the patients’ PCS and VT; (2) one significant actor effect: the patients’ anxiety on the patients’ MCS; and (3) one significant partner effect: the patients’ depression on the spouses’ VT.

## Discussion

To the best of our knowledge, the present study is the first attempt in mainland China to examine the HRQOL of both advanced cancer patients and their spousal caregivers. The findings of the study showed the following: (1) There were small to moderate effect sizes for the association between the patients’ HRQOL and that of their spouses (*r* = 0.193–0.398). (2) There were multiple independent factors influencing the HRQOL (SF-12 PCS, MCS, VT, and RE) of couples. These included: (i) patients’ characteristics: gender, length of time since the diagnosis of cancer; (ii) spousal caregivers’ characteristics: level of education, working status, and being informed about the disease; (iii) characteristics of dyads: improved dyadic relationship after the diagnosis of cancer; and (iv) the anxiety and depression (HADS) of the couples. Of all of these factors, anxiety and depression (PCFAC: HAD) had the most influence on the HRQOL (SF-12 PCS, MCS, VT, and RE) of both patients and spousal caregivers. Anxiety and depression (PCFAC: HAD) in patients was not only significantly associated with all four SF-12 domains (SF-12 PCS, MCS, VT, and RE) for the patients themselves, but significantly associated with the SF-12 MCS domain for spousal caregivers. (3) Anxiety and depression may have both actor and partner effects on the couples’ HRQOL to various degrees.

Based on the results of this study, the following three aspects are discussed: (i) the relationship between the HRQOL of the advanced cancer patients and that of their spousal caregivers; and (ii) the factors potentially contributing to the HRQOL of advanced cancer patients and their spousal caregivers; and (iii) the impact of anxiety and depression on HRQOL.

### The relationship between the HRQOL of cancer patients and that of their spousal caregivers

The findings of this study showed that significant correlations existed in all of the paired variables between the patients and their spousal caregivers, with the exception of the “role physical” of SF-12 (*r* = 0.193–0.398). These significant correlations may indicate a mutual impact between the cancer patients and their spousal caregivers in HRQOL. These correlations between patients and spousal caregivers in QOL were consistent with those found in other studies. A study showed that the total QOL score of the patients was associated with both the total score and the score for each dimension of the QOL of their spousal caregivers (*r* = 0.27–0.44) [4]. Another longitudinal study of patients with prostate cancer and their spouses also reported small to moderately significant correlations in QOL between the patients and their spouses over time (*r* = 0.25, 0.24, 0.23, and 0.23 at baseline, and at the 4-, 8-, and 12- month follow-ups respectively) [14].

The HRQOL of the couples coping with cancer in this study in Wuxi revealed that the PCS of the patients (37.70 + 9.49) and the spousal caregivers (43.05 + 9.13), and the MCS of the patients (41.92 + 7.18) and the spousal caregivers (42.25 + 7.70), were lower than those of a sample of the general population of urban Chengdu city (*n* = 1365) in China (PCS = 51.2 + 6.6, MCS = 49.9 + 7.7) [42]. The results also showed that spousal caregivers had higher scores than the patients in most of the subscales of SF-12 (with the exception of MCS, SF, and MH). This may be because the patients were suffering from the cancer and its treatment, particularly from the side-effects of chemotherapy. However, it should be noted that mental health was the only domain of SF-12 in which the spousal caregivers had lower scores than the patients, although no significant difference was identified. This finding may partly echo previous evidence indicating that the emotional distress of spousal caregivers may be as great as or even greater than that of the patients themselves [13, 43].

The finding in the present study that the RE score was the lowest among all of the SF-12 domains and dimensions is partly consistent with the finding of another study, which examined the HRQOL and related factors of Chinese patients undergoing chemotherapy for advanced cancer [23]. The HRQOL of cancer patients in that study was assessed using both the SF-36 and the European Organization for the Research and Treatment of Cancer’s quality of life questionnaire (EORTC QLQ-C30). The study also identified that RP and RE as measured by SF-36, and role functioning as measured by EORTC QLQ-C30 had the lowest scores among the domains of the instruments. These consistent findings on the scores on role functioning in cancer patients using different instruments should attract attention to this aspect of the QOL of cancer patients. Indeed, cancer and its treatment pose challenges to couples, in that they find it necessary to readjust and adapt to changes in both their family and occupational roles [6, 8, 9].

### The factors contributing to the HRQOL of cancer patients and spousal caregivers

Multiple independent factors contributing to HRQOL (SF-12 PCS, MCS, VT, and RE) were identified in the present analysis using multiple linear regressions, including those for gender, time since the diagnosis of cancer, level of education of the spousal caregivers, the spouses’ working status, the extent to which the spousal caregivers were informed about the disease, the dyadic relationship after the diagnosis of cancer, and anxiety and depression.

#### Patients’ characteristics

This study found that male patients (female spousal caregivers) had higher SF-12 VT scores. Evidence is accumulating that there are gender differences in how the partners in a couple experience coping with cancer [44]. A review that explored the experiences of spousal caregivers caring for cancer patients from the perspective of gender revealed that female caregivers of spouses with cancer experienced higher negative impacts in many different dimensions when compared to their male counterparts, such as poorer mental health, physical health, HRQOL, life satisfaction, and marital adjustment [45]. A study of cancer patients and spousal caregivers showed that female spousal caregivers of cancer patients are more likely to experience personal growth than males in terms of being open to new possibilities, relating to others, appreciating life, finding personal strength, and experiencing spiritual change [46].

One reason for the differences between female and male caregivers may lie in the differences in gender role commitment [47]. Female caregivers of cancer patients have been found to position themselves as all-encompassing expert caregivers, taking on too many responsibilities and sacrificing themselves, leading to distress, in contrast to male caregivers who see caring as a task of competency (not their traditional role), leading to feelings of self-mastery or satisfaction [48, 49]. Female caregivers are traditionally more likely to perform personal care in relation to family members and household chores, which may be more demanding and ongoing than traditional male tasks [48].

A longer duration since the diagnosis of cancer was found to be associated with lower SF-12 VT scores for patients. This finding was inconsistent with another study, which found no significant association between the diagnosis and duration of cancer and the wellbeing of caregivers [50].

#### Spousal caregivers’ characteristics

The characteristics of spousal caregivers, such as level of education, working status, and being informed about the disease, were identified as being related to the HRQOL of the patients. These findings again indicate that there is a mutual impact between cancer patients and spousal caregivers with regard to their HRQOL. No similar quantitative study on this aspect was identified. This was supported by the findings of a focus group study on the experiences of Chinese couples living with cancer. The findings showed that providing the patients and/or their spousal caregivers with accurate information about their illness, as well as an appropriate amount of information and at the right time, could improve their experience of living with cancer [51].

#### Dyad characteristics

In this study, about one-third (29.0 %) of the couples (87.8 %) reported that their relationship with their partner improved after the diagnosis of cancer. This relationship dynamic was echoed in a literature review, which found that caregivers reported that their relationship with the care-receiver had improved because of the caregiving process [52]. The caregivers experienced stronger feelings of love and of being closer together, resulting in an enhanced and deeper relationship with the care-receiver [53, 54].

The results of this study also showed that an improved dyadic relationship after the diagnosis of cancer was associated with a lower score for SF-12 RE in both patients and spousal caregivers, and a higher score for SF-12 VT in spousal caregivers. The association between improved dyadic relationship and lower role emotional (RE) pointed to the fact that couples with an improved dyadic relationship were able to use problem-focused coping and/or meaning-focused coping, rather than emotion-focused coping. This demonstrated that these couples were coping effectively, thus leading to the higher VT. A framework on the positive aspects of caregiving illustrated that an improved relationship between caregivers and care-receivers with dementia (spousal caregivers and patients with cancer in this case) could benefit the wellbeing of the caregivers and support their continued involvement in the caregiving process [55].

The association between the QOL of a couple and their relationship with each other was also supported by other studies. For instance, the level of marital satisfaction was found to be one of the strongest factors influencing whether a spousal caregiver would have problems performing the caregiving role [13]; and dyadic adjustment was associated with a spouse’s mood disturbances (*r* = −0.49, *P* = 0.001) and mental health functioning (*r* = 0.35, *P* = 0.02) [56]. Both the mental (*r* = 0.33, *P* < 0.05) and physical (*r* = 0.28, *P* < 0.05) health of patients was positively related to their caregiver’s marital satisfaction [57]. This finding is a reminder that improving the relationship of couples may be an effective method of enhancing the couples’ HRQOL in their journey of coping with cancer together. Indeed, a review of spousal couple-based intervention studies revealed that intervention studies focusing on marital/family care showed positive outcomes, including improvements in communication, dyadic coping, and in the QOL of both the patients and their partners [58].

#### Anxiety and depression

Anxiety and depression were identified as the strongest independent factors influencing the HRQOL (SF-12 PCS, MCS, VT, and RE) of both the patients and spousal caregivers in this study. A high level of anxiety and depression (PCFAC: HAD) in spousal caregivers was significantly associated with all of the four SF-12 scores (PCS, MCS, VT, and RE) of the spousal caregivers. A high level of anxiety and depression (PCFAC: HAD) in patients was also significantly associated with all of the four SF-12 domains (PCS, MCS, VT, and RE) of the patients and with the SF-12 MCS domain of their spousal caregivers. These findings are consistent with those of previous studies, in that higher levels of anxiety and depression have generally been associated with poorer HRQOL in cancer patients [59–61]. An intervention study has reported that efforts to manage depression had a positive impact on improving the HRQOL of cancer patients [62].

### Impact of anxiety and depression on HRQOL

An APIM analysis showed that all of the four sub-models resulted in convergence and showed goodness of fit. These findings support the view that anxiety directly or indirectly through depression impacted on the SF-12 domains as actor effects and/or as partner effects in the four detected models. These findings also support the argument that couples have a mutual impact on each other in their journey of coping with cancer together.

In addition, the general negative impact of anxiety and depression on the couples’ HRQOL as revealed in the APIM analysis further supports and is consistent with the findings from the multiple linear regressions, in that anxiety and depression were the strongest independent factors influencing the HRQOL (SF-12 PCS, MCS, VT, and RE) of both the patients and their spousal caregivers. However, it is worth noting that only one significant actor effect (patients’ anxiety on patients’ MCS), and one significant partner effect (patients’ depression on spouses’ VT) were identified. There were five exceptions to the trend of positive impacts on partner effects, including the impact of the patients’ anxiety on their spouses’ PCS, MCS, and RE; and the impact of the spouses’ depression on the patients’ PCS, and VT (Table 5). These findings revealed that, to a certain extent, the distress of the couples, e.g., the patients’ anxiety and their spouses’ depression, may motivate the couples to cope with cancer as dyads, or have a positive impact on the couples’ HRQOL. These findings also indicated that the impact of both the patients’ and their spouses’ anxiety and depression on the couples’ HRQOL is complicated and deserves further exploration, particularly at the dyadic level.

In summary, the results of this study showed that the couples’ HRQOL was relatively low, and that there was a mutual impact between the cancer patients and their spousal caregivers in various aspects of HRQOL. The factors that were identified as possibly affecting the HRQOL of couples can be grouped into three main aspects: (1) individual characteristics of the patients and their spousal caregivers: male patients, female spousal caregivers, shorter duration since the diagnosis of cancer, lower level of education of spousal caregivers, spouses who hold a full-time job, spouses who are partly informed about the disease; (2) dyadic characteristic: improved dyadic relationship after the diagnosis of cancer; and (3) the couples’ distress: a lower level of anxiety and depression in both the cancer patients and their spouses. The multiple factors that were identified as influencing the couples’ HRQOL provide valuable evidence of the components that need to be included in any intervention to improve the HRQOL of couples. Health care professionals, when working with couples coping with cancer, should pay special attention not only to the HRQOL of cancer patients, but also to providing support to their spousal caregivers, who are accompanying their loved ones through the cancer trajectory. To improve the HRQOL of couples in cancer practice, clinicians need to pay more attention to multiple aspects, including to developing and delivering the intervention as early as possible, to paying special attention to the characteristics of both the patients and their spouses, to enhancing the couple’s relationship, and to helping couples to manage their distress, e.g., anxiety or depression.

### Strengths and limitations

The main strength of the present study was the dyadic data obtained from both the cancer patients and their spousal caregivers. The dyadic data made it possible to explore the mutual impact between the couples; and it also made it possible to analyze factors of potential impact from different aspects, including the characteristics of the patients, spousal caregivers, and dyads.

The findings of the study contribute to the understanding of the HRQOL of Chinese couples in their journey of coping with cancer as a dyad; however, it is not without limitations. The study was limited by its cross-sectional design, and the conclusions that were drawn need to be confirmed in future studies. Moreover, the fact that the study population was comprised of patients with advanced cancer undergoing chemotherapy in an oncology hospital in Wuxi, China, may mean that the results are not generalizable to couples in other stages of cancer, out-patients, and those living in other countries. Also, the heterogeneity of the subjects, who suffered from different types of cancer, might have affected the attempt to measure levels of HRQOL. A large sample study on the impact of different types of cancer and different stages of cancer on HRQOL is needed.

In addition, this study contained a relatively small sample for testing APIM models, barely meeting the critical minimum number for the target population to be tested for medium to large effects using APIM [63]. One should therefore interpret the results of this study with caution. Until this study is replicated with larger samples, its findings can only be taken as suggestive [64], p. 72). Future studies involving larger sample sizes for APIM analysis are highly recommended.

Another limitation lies in the fact that the HRQOL of couples coping with cancer was measured using the SF-12. As a generic measure, the SF-12 might not be sufficiently sensitive or specific to evaluate the HRQOL of advanced cancer patients and spousal caregivers. The application of both generic and specific measures of HRQOL for couples coping with cancer is needed. However, the choice of SF-12 fits the aim in this study of assessing the QOL of both the patients and their spousal caregivers. It also made it possible to compare the HRQOL outcomes of the two parties.

## Conclusion

Notwithstanding these limitations, the findings of this study call for attention to be paid to HRQOL and its related factors for couples coping with cancer as dyads. It highlights the following as areas in which couples coping with advanced cancer could benefit from interventions to improve their HRQOL: individual characteristics of both the cancer patients and their spouses, their marital relationship, and their anxiety and depression. Indeed, the evidence showed that interventions to improve the marital relationship of the couples [58] and their ability to manage depression [62] were effective at improving the HRQOL of couples or cancer patients. In addition, the mutual impact between the cancer patients and their spousal caregivers should alert clinicians to the importance of treating couples as a dyad when delivering interventions to improve the HRQOL of patients and spouses. The complex impact of anxiety and depression on the HRQOL of couples is an area that deserves further investigation in cancer practice.

## Abbreviations

BP, bodily pain; GH, general health; HADS, hospital anxiety and depression scale; HRQOL, health related quality of life; MCS, the Mental Component Summary; MH, mental health; PCS, the Physical Component Summary; PF, physical functioning; RE, role-emotional; RP, role-physical; SEM, structural equation modeling; SF, social functioning; SF-12, the Medical Outcomes Study 12-item Short Form; VT, vitality

## Additional files

Additional file 1: Table S1. Association between the variables of both the patients and their spousal caregivers and the PCS, MCS, VT, and RE domains of the SF-12 of the patients. **Table S2.** Association between the variables of both the patients and spousal caregivers and the PCS, MCS, VT, and RE domains of the SF-12 of the spousal caregivers. (DOC 293 kb) Additional file 2: Figure S1. Theoretical model in testing the impact of anxiety and depression on Health related quality of life. **Figure S2.** Four sub-models (sub-model 1-4) for testing testing the impact of anxiey and depression on HRQOL. (DOC 475 kb)
