# Supplementary material for: Factors influencing the health-related quality of life of Chinese advanced cancer patients and their spousal caregivers: a cross-sectional study
Source: BMC Palliat Care. 2016 Aug 2;15:72. doi: 10.1186/s12904-016-0142-3 (PMC4971682; doi:10.1186/s12904-016-0142-3)
Supplement: Additional file 1: Table S1. — Association between the variables of both the patients and their spousal caregivers and the PCS, MCS, VT, and RE domains of the SF-12 of the patients. Table S2. Association between the variables of both the patients and spousal caregivers and the PCS, MCS, VT, and RE domains of the SF-12 of the spousal caregivers. (DOC 293 kb) [file 12904_2016_142_MOESM1_ESM.doc]

Note: SD=standard deviation; P=Patients; SC=Spousal Caregivers; PCS=Physical Component Summary; MCS=Mental Component Summary, VT=Vitality; and RE=Role Emotional; HADS=Hospital Anxiety and Depression Scales.

| **Table S1**  Association between the variables of both the patients and their spousal caregivers and the PCS, MCS, VT, and RE domains of the SF-12 of the patients | | | | | | | | | | | | | |
| --- | --- | --- | --- | --- | --- | --- | --- | --- | --- | --- | --- | --- | --- |
| **Variables** | n | Physical Component Summary | | | Mental Component Summary | | | Vitality | | | Role Emotional | | |
|  |  | Mean(SD) | r/t/F* | P-value | Mean(SD) | r/t/F* | P-value | Mean(SD) | r/t/F* | P-value | Mean(SD) | r/t/F* | P-value |
| **S1a: Patients’ characteristics** | |  |  |  |  |  |  |  |  |  |  |  |  |
| Age_P | 131 | 37.70(9.46) | 0.025 | 0.774 | 41.92(7.18) | 0.008 | 0.924 | 44.75(8.89) | 0.020 | 0.820 | 34.78(10.68) | -0.013 | 0.881 |
| Gender** Male | 82 | 38.42(9.76) |  |  | 42.04(7.23) |  |  | 46.40(8.77) |  |  | 34.46(10.50) |  |  |
| Female | 49 | 36.49(8.90) | 1.133 | 0.259 | 41.72(7.15) | 0.243 | 0.808 | 42.00(8.47) | 2.814 | 0.006 | 35.31(11.07) | -0.438 | 0.662 |
| Level of education_P | | |  |  |  |  |  |  |  |  |  |  |  |
| Primary school or less | 64 | 37.75(9.58) |  |  | 42.21(7.47) |  |  | 45.71(9.85) |  |  | 36.07(10.44) |  |  |
| High school | 49 | 38.19(9.75) |  |  | 41.83(6.31) |  |  | 44.46(8.06) |  |  | 34.74(10.75) |  |  |
| University or above | 18 | 36.15(8.56) | 0.306 | 0.737 | 41.13(8.58) | 0.161 | 0.851 | 42.16(7.09) | 1.163 | 0.316 | 30.30(10.70) | 2.090 | 0.128 |
| Working status-P |  |  |  |  |  |  |  |  |  |  |  |  |  |
| Working | 75 | 37.35(8.77) |  |  | 42.60(6.87) |  |  | 45.60(8.80) |  |  | 34.53910.55) |  |  |
| Not working | 56 | 38.17(10.38) | -0.488 | 0.626 | 41.01(7.75) | 1.255 | 0.212 | 43.62(8.95) | 1.268 | 0.207 | 35.11(10.94) | -0.305 | 0.761 |
| Time since diagnosis | 131 | 37.70(9.46) | -0.062 | 0.484 | 41.92(7.18) | -0.140 | 0.111 | 44.75(8.89) | -0.206 | 0.018 | 34.78(10.68) | -0.110 | 0.209 |
| Informed about the disease _P | |  |  |  |  |  |  |  |  |  |  |  |  |
| Partly informed | 33 | 35.74(9.06) |  |  | 40.14(5.58) |  |  | 45.01(6.78) |  |  | 32.02(8.67) |  |  |
| Well informed | 98 | 38.36(9.55) | -1.382 | 0.169 | 42.52(7.57) | -1.655 | 0.100 | 44.67(9.52) | 0.187 | 0.852 | 35.71(11.16) | -1.731 | 0.086 |
| Types of cancer |  |  |  |  |  |  |  |  |  |  |  |  |  |
| Digestive system cancer | 72 | 36.36(8.24) |  |  | 41.62(6.26) |  |  | 44.54(7.89) |  |  | 33.71(9.57) |  |  |
| Lung cancer | 35 | 38.67(11.93) |  |  | 42.84(9.33) |  |  | 46.02(10.49) |  |  | 36.75(13.10) |  |  |
| Urogenital system cancer | 19 | 40.12(8.27) |  |  | 40.8796.84) |  |  | 41.9299.07) |  |  | 34.60(10.25) |  |  |
| Others | 5 | 40.97(10.26) | 1.228 | 0.302 | 43.68(3.05) | 0.459 | 0.711 | 49.76(8.42) | 1.439 | 0.234 | 37.07(9.36) | 0.709 | 0.548 |
| **S1b: Spousal caregivers’ characteristics** | | |  |  |  |  |  |  |  |  |  |  |  |
| Age_SC | 131 | 37.70(9.46) | 0.016 | 0.857 | 41.92(7.18) | -0.052 | 0.554 | 44.75(8.89) | -0.033 | 0.710 | 34.78(10.68) | -00.042 | 0.633 |
| Level of education_SC |  |  |  |  |  |  |  |  |  |  |  |  |  |
| Primary school or less | 67 | 38.55(9.25) |  |  | 43.50(7.72) |  |  | 46.85(9.34) |  |  | 36.97(10.58) |  |  |
| High school | 49 | 36.65(10.33) |  |  | 40.82(6.47) |  |  | 43.23(7.97) |  |  | 33.60(10.68) |  |  |
| University or above | 15 | 37.31(7.42) | 0.580 | 0.561 | 38.46(4.98) | 4.134 | 0.018 | 40.37(7.08) | 4.652 | 0.011 | 28.87(8.68) | 4.202 | 0.017 |
| Working status-SC |  |  |  |  |  |  |  |  |  |  |  |  |  |
| Working | 69 | 38.61(8.80) |  |  | 42.75(7.09) |  |  | 46.15(7.84) |  |  | 36.06(9.98) |  |  |
| Not working | 62 | 36.69(10.12) | 1.161 | 0.248 | 40.99(7.21) | 1.403 | 0.163 | 43.21(9.76) | 1.910 | 0.058 | 33.35(11.31) | 1.457 | 0.148 |
| Informed about the disease _SC | | |  |  |  |  |  |  |  |  |  |  |  |
| Partly informed | 24 | 37.54(7.76) |  |  | 42.24(8.25) |  |  | 46.91(8.35) |  |  | 36.51(10.94) |  |  |
| Well informed | 107 | 37.73(9.83) | -0.091 | 0.927 | 41.85(6.95) | 0.240 | 0.811 | 44.27(8.97) | 1.320 | 0.189 | 34.39(10.63) | 0.877 | 0.382 |
| Time spent by spousal caregivers in caring for patients / day | | | | | |  |  |  |  |  |  |  |  |
| <2hours | 10 | 44.45(10.24) |  |  | 45.90(5.72) |  |  | 48.76(12.05) |  |  | 43.22(10.56) |  |  |
| 2～4hours | 14 | 42.46(7.40) |  |  | 44.36(9.39) |  |  | 47.75(6.84) |  |  | 40.10(11.15) |  |  |
| >4hours～6hours | 25 | 35.20(7.90) |  |  | 41.05(4.52) |  |  | 43.72(8.22) |  |  | 31.03(10.22) |  |  |
| >6hours～8hours | 21 | 39.81(10.27) |  |  | 40.99(7.62) |  |  | 44.40(8.01) |  |  | 35.58(9.24) |  |  |
| >8hours | 61 | 35.79(9.25) | 3.766 | 0.006 | 41.38(7.42) | 1.458 | 0.219 | 43.96(9.22) | 1.125 | 0.348 | 33.44(10.33) | 3.767 | 0.006 |
| **S1c: Dyads’ characteristics** | |  |  |  |  |  |  |  |  |  |  |  |  |
| Duration of Marriage | 131 | 37.70(9.46) | 0.029 | 0.741 | 41.92(7.18) | -0.007 | 0.936 | 44.75(8.89) | -0.003 | 0.974 | 34.78(10.68) | -00.013 | 0.879 |
| Relationship with their partner before the diagnosis of cancer | | | | |  |  |  |  |  |  |  |  |  |
| Very good | 115 | 37.49(9.57) |  |  | 41.84(7.40) |  |  | 44.86(9.02) |  |  | 34.49(10.88) |  |  |
| Normal | 16 | 39.19(8.79) | -0.671 | 0.503 | 42.49(5.42) | -0.341 | 0.734 | 43.98(8.11) | 0.372 | 0.710 | 36.86(9.12) | -0.830 | 0.408 |
| Change in the relationship with their partner after the diagnosis of cancer | | | | | |  |  |  |  |  |  |  |  |
| Getting better | 38 | 37.88(10.21) |  |  | 41.69(7.23) |  |  | 46.69(8.90) |  |  | 32.83(10.03) |  |  |
| No change | 93 | 37.62(9.20) | 0.143 | 0.887 | 42.01(7.17) | -0.228 | 0.820 | 43.96(8.77) | 1.604 | 0.111 | 35.58(10.89) | -1.340 | 0.183 |
| Financial burden on the family due to the treatment of cancer | | | | | |  |  |  |  |  |  |  |  |
| Serious | 77 | 36.43(9.11) |  |  | 41.03(6.13) |  |  | 44.22(8.45) |  |  | 32.99(9.99) |  |  |
| Normal | 49 | 39.15(9.56) |  |  | 43.39(7.92) |  |  | 45.90(9.34) |  |  | 37.48(10.73) |  |  |
| Mild or None | 5 | 42.96(12.04) | 2.073 | 0.130 | 41.07(12.80) | 1.670 | 0.192 | 41.71(11.47) | 0.838 | 0.435 | 35.95(16.56) | 2.752 | 0.068 |
| **S1d: HADS** | | |  |  |  |  |  |  |  |  |  |  |  |
| Anxiety_P | 131 | 37.70(9.46) | -0.372 | 0.000 | 41.92(7.18) | -0.408 | 0.000 | 44.75(8.89) | -0.265 | 0.002 | 34.78(10.68) | -0.415 | 0.000 |
| Depression_P | 131 | 37.70(9.46) | -0.373 | 0.000 | 41.92(7.18) | -0.353 | 0.000 | 44.75(8.89) | -0.291 | 0.001 | 34.78(10.68) | -0.382 | 0.000 |
| Anxiety_SC | 131 | 37.70(9.46) | -0.259 | 0.003 | 41.92(7.18) | -0.306 | 0.000 | 44.75(8.89) | -0.204 | 0.019 | 34.78(10.68) | -0.358 | 0.000 |
| Depression_SC | 131 | 37.70(9.46) | -0.246 | 0.005 | 41.92(7.18) | -0.313 | 0.000 | 44.75(8.89) | -0.200 | 0.022 | 34.78(10.68) | -0.336 | 0.000 |

* r/t/F: r=one group using person correlation; t=two groups using t-test; F=above two groups using one-way ANOVA.

** Gender: Given that the participants included in this study were cancer couples, only the gender of the patients was analyzed.

| **Table S2** Association between the variables of both the patients and spousal caregivers and the PCS, MCS, VT, and RE domains of the SF-12 of the spousal caregivers | | | | | | | | | | | | | |
| --- | --- | --- | --- | --- | --- | --- | --- | --- | --- | --- | --- | --- | --- |
| **Variables** | n | Physical Component Summary | | | Mental Component Summary | | | Vitality | | | Role Emotional | | |
|  |  | Mean(SD) | r/t/F* | P-value | Mean(SD) | r/t/F* | P-value | Mean(SD) | r/t/F* | P-value | Mean(SD) | r/t/F* | P-value |
| **S2a: Patients’ characteristics** | |  |  |  |  |  |  |  |  |  |  |  |  |
| Age_P | 131 | 43.05(9.13) | -0.106 | 0.227 | 42.25(7.70) | -0.002 | 0.986 | 47.21(9.61) | -0.040 | 0.652 | 38.11(9.95) | -0.054 | 0.540 |
| Gender** Male | 82 | 42.84(9.07) |  |  | 42.16(7.70) |  |  | 47.50(9.35) |  |  | 38.08(9.13) |  |  |
| Female | 49 | 43.38(9.31) | -0.323 | 0.747 | 42.41(7.77) | -0.172 | 0.863 | 46.72(10.11) | 0.449 | 0.654 | 38.16(11.30) | -0.048 | 0.962 |
| Level of education_P | | |  |  |  |  |  |  |  |  |  |  |  |
| Primary school or less | 64 | 42.75(9.28) |  |  | 41.61(7.44) |  |  | 46.65(10.47) |  |  | 38.26(10.82) |  |  |
| High school | 49 | 43.94(8.15) |  |  | 42.44(7.33) |  |  | 48.37(8.32) |  |  | 37.02(7.65) |  |  |
| University or above | 18 | 41.70(11.22) | 0.458 | 0.634 | 44.04(9.61) | 0.719 | 0.489 | 46.07(9.91) | 0.585 | 0.558 | 40.55(12.21) | 0.837 | 0.435 |
| Working status-P |  |  |  |  |  |  |  |  |  |  |  |  |  |
| Working | 75 | 43.13(9.05) |  |  | 42.63(7.66) |  |  | 47.48(9.78) |  |  | 38.26(9.93) |  |  |
| Not working | 56 | 42.94(9.32) | 0.113 | 0.910 | 41.75(7.80) | 0.646 | 0.519 | 46.85(9.45) | 0.370 | 0.712 | 37.91(10.07) | 0.201 | 0.841 |
| Time since diagnosis | 131 | 43.05(9.13) | -0.055 | 0.531 | 42.25(7.70) | 0.004 | 0.968 | 47.21(9.61) | -0.017 | 0.846 | 38.11(9.95) | -0.018 | 0.837 |
| Informed about the disease _P | |  |  |  |  |  |  |  |  |  |  |  |  |
| Partly informed | 33 | 40.67(7.67) |  |  | 41.65(6.82) |  |  | 47.14(9.05) |  |  | 37.27(8.42) |  |  |
| Well informed | 98 | 43.85(9.47) | -1.744 | 0.084 | 42.46(8.00) | -0.520 | 0.604 | 47.24(9.84) | -0.050 | 0.960 | 38.39(10.42) | -0.558 | 0.578 |
| Types of cancer |  |  |  |  |  |  |  |  |  |  |  |  |  |
| Digestive system cancer | 72 | 42.52(8.93) |  |  | 41.67(7.45) |  |  | 46.35(9.45) |  |  | 38.06(10.03) |  |  |
| Lung cancer | 35 | 43.68(9.34) |  |  | 42.55(8.22) |  |  | 50.05(10.09) |  |  | 38.03(9.11) |  |  |
| Urogenital system cancer | 19 | 42.58(9.13) |  |  | 44.19(8.60) |  |  | 46.16(9.04) |  |  | 37.24(10.41) |  |  |
| Others | 5 | 48.12(11.63) | 0.662 | 0.577 | 41.2893.45) | 0.578 | 0.630 | 43.72(9.00) | 1.522 | 0.212 | 42.66(14.58) | 0.392 | 0.759 |
| **S2b: Spousal caregivers’ characteristics** | | |  |  |  |  |  |  |  |  |  |  |  |
| Age_SC | 131 | 43.05(9.13) | -0.088 | 0.318 | 42.25(7.70) | 0.022 | 0.803 | 47.21(9.61) | -0.043 | 0.623 | 38.11(9.95) | -0.036 | 0.686 |
| Level of education_SC |  |  |  |  |  |  |  |  |  |  |  |  |  |
| Primary school or less | 67 | 43.57(8.89) |  |  | 42.68(8.20) |  |  | 47.90(9.51) |  |  | 38.39(9.89) |  |  |
| High school | 49 | 42.32(9.60) |  |  | 41.60(6.57) |  |  | 46.11(9.49) |  |  | 37.94(9.92) |  |  |
| University or above | 15 | 43.09(9.08) | 0.265 | 0.767 | 42.50(9.13) | 0.284 | 0.754 | 47.75(10.76) | 0.515 | 0.599 | 37.44(11.12) | 0.066 | 0.936 |
| Working status-SC |  |  |  |  |  |  |  |  |  |  |  |  |  |
| Working | 69 | 43.54(8.93) |  |  | 42.56(7.83) |  |  | 47.46(10.35) |  |  | 39.06(9.75) |  |  |
| Not working | 62 | 42.50(9.39) | 0.654 | 0.514 | 41.91(7.62) | 0.482 | 0.631 | 46.94(8.79) | 0.308 | 0.759 | 37.05(10.15) | 1.156 | 0.250 |
| Informed about the disease _SC | | |  |  |  |  |  |  |  |  |  |  |  |
| Partly informed | 24 | 40.64(9.19) |  |  | 42.24(7.91) |  |  | 46.91(9.34) |  |  | 34.88(9.03) |  |  |
| Well informed | 107 | 43.59(9.07) | -1.434 | 0.154 | 42.26(7.70) | -0.008 | 0.994 | 47.28(9.71) | -0.169 | 0.866 | 38.83(10.05) | -1.774 | 0.078 |
| Time spent by spousal caregivers in caring for patients / day | | | | | |  |  |  |  |  |  |  |  |
| <2hours | 10 | 47.72(9.87) |  |  | 47.22(9.33) |  |  | 51.77(12.73) |  |  | 42.66(12.97) |  |  |
| 2～4hours | 14 | 43.0397.85) |  |  | 43.81(6.43) |  |  | 47.75(7.89) |  |  | 40.90(6.37) |  |  |
| >4hours～6hours | 25 | 41.85(9.73) |  |  | 44.34(8.08) |  |  | 50.57(10.28) |  |  | 38.19(7.91) |  |  |
| >6hours～8hours | 21 | 44.15(8.02) |  |  | 39.58(7.90) |  |  | 45.83(8.19) |  |  | 37.44(10.80) |  |  |
| >8hours | 61 | 42.40(9.40) | 0.913 | 0.459 | 41.15(7.03) | 2.718 | 0.033 | 45.44(9.26) | 2.024 | 0.095 | 36.92(10.48) | 1.040 | 0.389 |
| **S2c: Dyads’ characteristics** | |  |  |  |  |  |  |  |  |  |  |  |  |
| Duration of Marriage | 131 | 43.05(9.13) | -0.052 | 0.553 | 42.25(7.70) | 0.010 | 0.911 | 47.21(9.61) | -0.030 | 0.730 | 38.11(9.95) | -0.036 | 0.685 |
| Relationship with their partner before the diagnosis of cancer | | | |  |  |  |  |  |  |  |  |  |  |
| Very good | 115 | 43.20(9.17) |  |  | 42.00(8.04) |  |  | 46.70(9.74) |  |  | 37.80(10.08) |  |  |
| Normal | 16 | 41.98(9.10) | 0.499 | 0.619 | 44.11(4.39) | -1.589 | 0.122 | 50.89(7.98) | -1.646 | 0.102 | 40.35(8.95) | -1.053 | 0.305 |
| Change in the relationship with their partner after the diagnosis of cancer | | | | | |  |  |  |  |  |  |  |  |
| Getting better | 38 | 44.40(10.73) |  |  | 42.38(8.58) |  |  | 51.46(7.91) |  |  | 36.36(10.30) |  |  |
| No change | 93 | 42.50(8.39) | 1.082 | 0.281 | 42.20(7.36) | 0.116 | 0.908 | 45.48(9.74) | 3.356 | 0.001 | 38.82(9.78) | -1.288 | 0.200 |
| Financial burden on the family due to the treatment of cancer | | | | | |  |  |  |  |  |  |  |  |
| Serious | 77 | 41.74(9.49) |  |  | 41.34(7.70) |  |  | 46.31(9.76) |  |  | 35.96(8.90) |  |  |
| Normal | 49 | 44.15(8.10) |  |  | 43.70(7.25) |  |  | 48.16(9.63) |  |  | 40.90(10.52) |  |  |
| Mild or None | 5 | 52.4597.17) | 3.971 | 0.021 | 42.15(11.40) | 1.415 | 0.247 | 51.77(5.51) | 1.142 | 0.323 | 43.78(12.75) | 4.791 | 0.010 |
| **S2d: HADS** | |  |  |  |  |  |  |  |  |  |  |  |  |
| Anxiety_P | 131 | 43.05(9.13) | -0.264 | 0.002 | 42.25(7.70) | -0.411 | 0.000 | 47.21(9.61) | -0.182 | 0.037 | 38.11(9.95) | -0.333 | 0.000 |
| Depression_P | 131 | 43.05(9.13) | -0.283 | 0.001 | 42.25(7.70) | -0.407 | 0.000 | 47.21(9.61) | -0.273 | 0.002 | 38.11(9.95) | -0.346 | 0.000 |
| Anxiety_SC | 131 | 43.05(9.13) | -0.379 | 0.000 | 42.25(7.70) | -0.432 | 0.000 | 47.21(9.61) | -0.294 | 0.000 | 38.11(9.95) | -0.387 | 0.000 |
| Depression_SC | 131 | 43.05(9.13) | -0.404 | 0.000 | 42.25(7.70) | -0.441 | 0.000 | 47.21(9.61) | -0.355 | 0.000 | 38.11(9.95) | -0.373 | 0.000 |

Note: SD=standard deviation; P=Patients; SC=Spousal Caregivers; PCS=Physical Component Summary; MCS=Mental Component Summary, VT=Vitality; and RE=Role Emotional; HADS=Hospital Anxiety and Depression Scales.

* r/t/F: r=one group using person correlation; t=two groups using t-test; F=above two groups using one-way ANOVA.

** Gender: Given that the participants included in this study were cancer couples, only the gender of the patients was analyzed.
