# Supplementary material for: Factors influencing the health-related quality of life of Chinese advanced cancer patients and their spousal caregivers: a cross-sectional study
Source: BMC Palliat Care. 2016 Aug 2;15:72. doi: 10.1186/s12904-016-0142-3 (PMC4971682; doi:10.1186/s12904-016-0142-3)
Supplement: Additional file 2: Figure S1. — Theoretical model in testing the impact of anxiety and depression on Health related quality of life. Figure S2. Four sub-models (sub-model 1-4) for testing testing the impact of anxiey and depression on HRQOL. (DOC 475 kb) [file 12904_2016_142_MOESM2_ESM.doc]

**Figure S1.** Theoretical model in testing the impact of anxiety and depression on Health related quality of life.

**Legends:**

SF-12 Domains were replaced by the M1, M2, M3, and M4 respectively in four different models as indicated in the Figure.

A-a, A-b, A-c, A-d, A-d stands for Actor effects;

P-a, P-b, P-c, P-d stands for Partner effects;

P=Patients, SC=Spousal Caregivers

**Figure S2. Four sub-models (sub-model 1-4) for testing testing the impact of anxiey and depression on HRQOL**


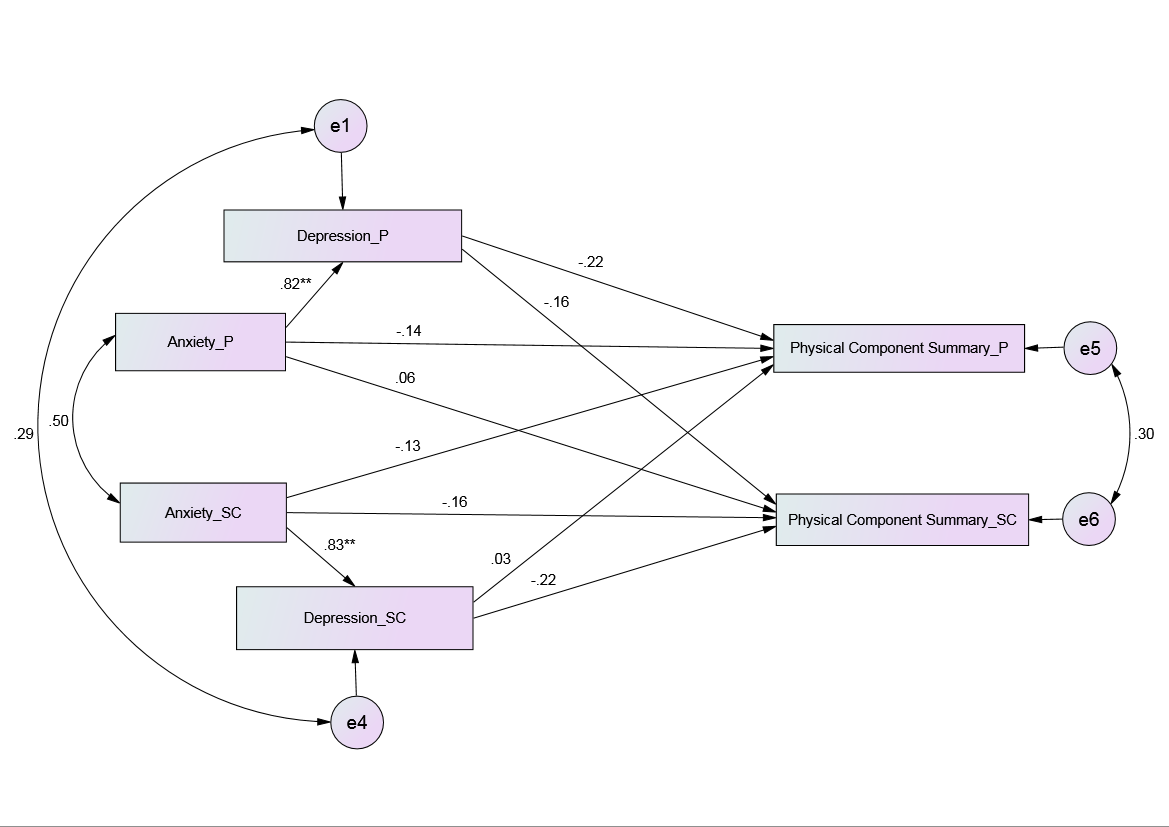


*Sub-Model 1 PCS*

***P<0.01*

**P<0.05; **P<0.01*

*Sub-Model 2 MCS*


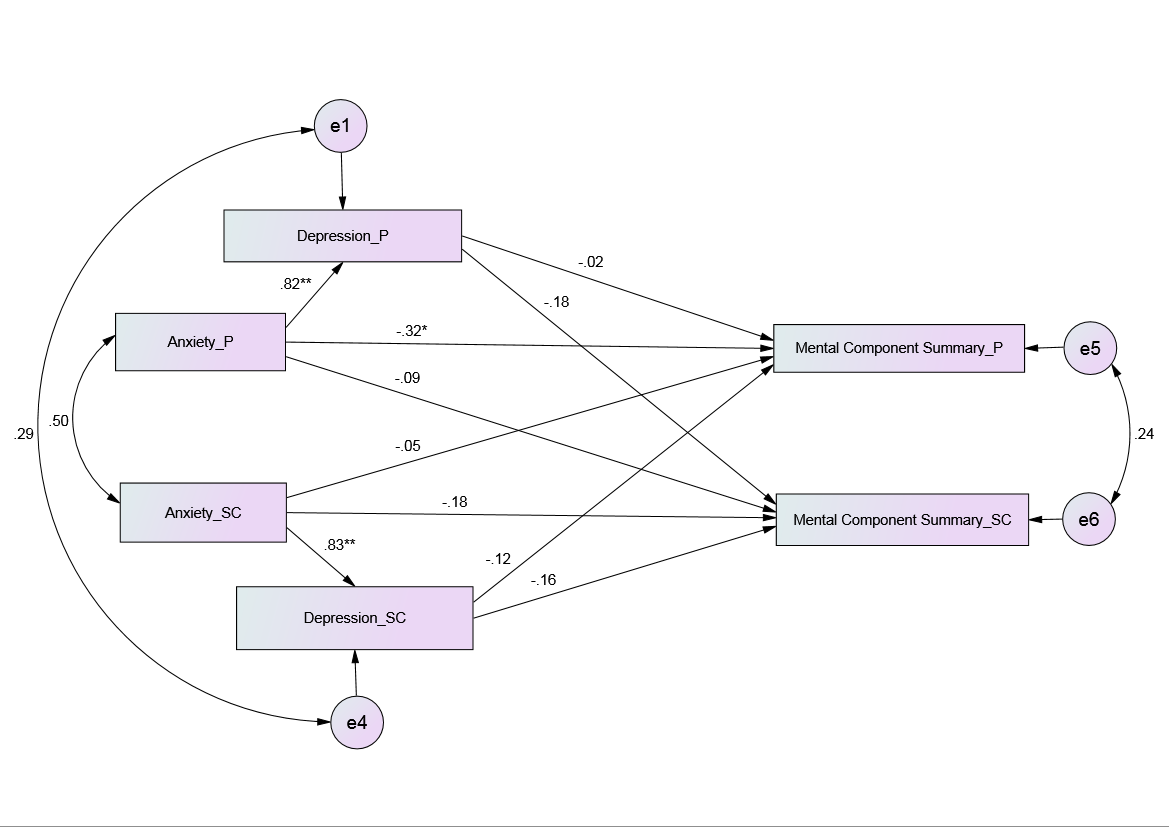


**P<0.05; **P<0.01*


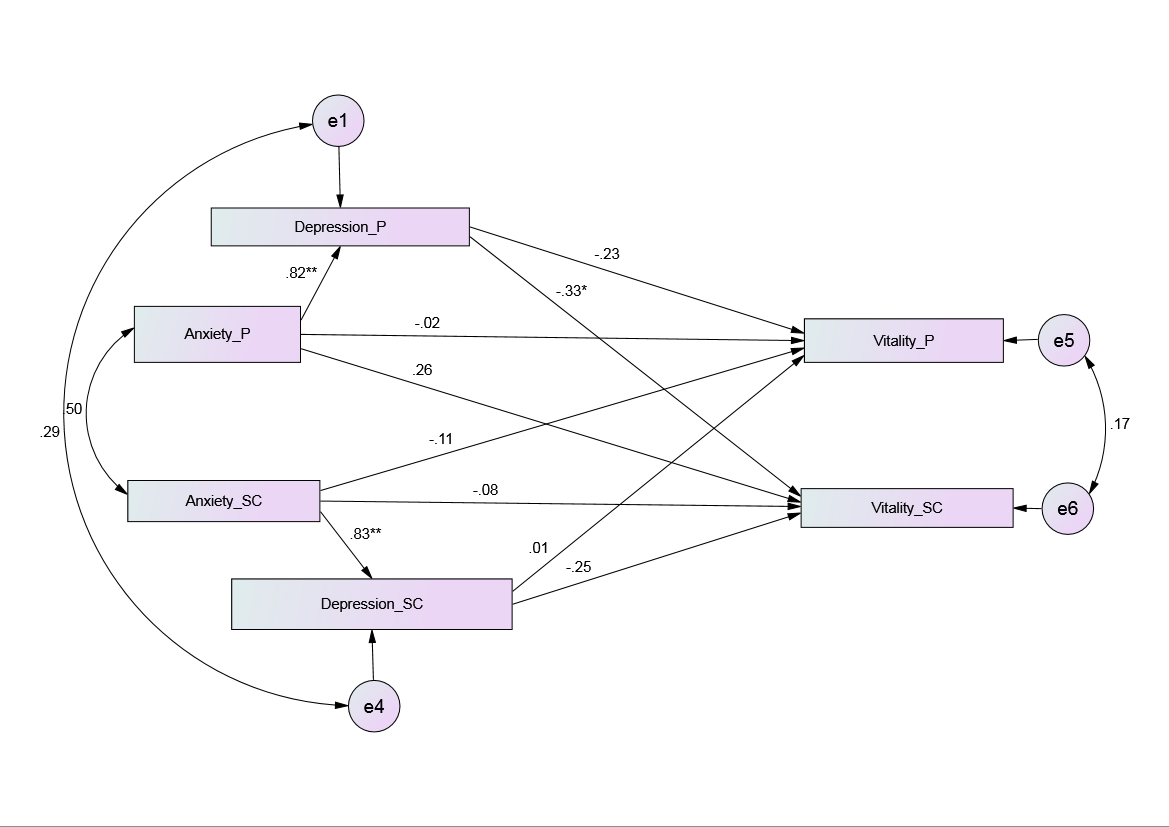


*Sub-Model 3 Vitality*

**P<0.05; **P<0.01*


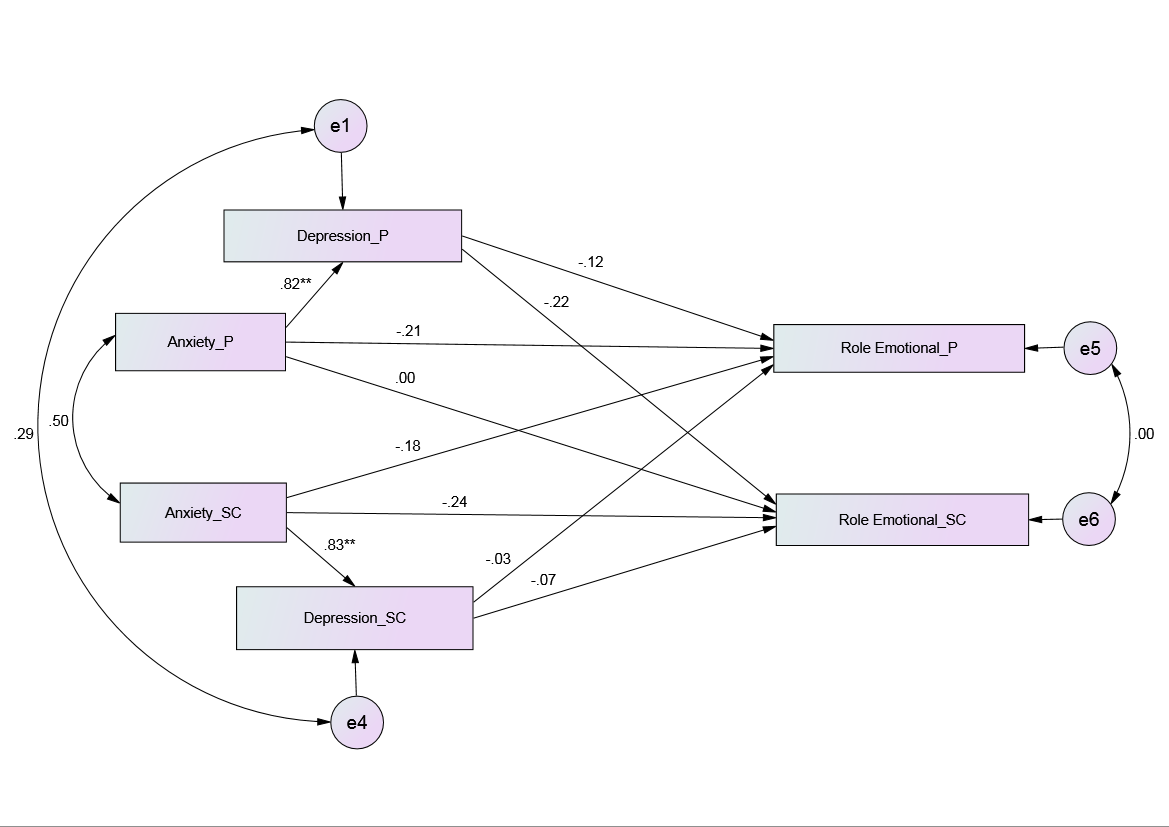


*Sub-Model 4 Role emotional*

***P<0.01*

**P<0.05; **P<0.01*
